# Associating Compositional, Nutritional and Techno-Functional Characteristics of Faba Bean (*Vicia faba* L.) Protein Isolates and Their Production Side-Streams with Potential Food Applications

**DOI:** 10.3390/foods12050919

**Published:** 2023-02-21

**Authors:** Magdalena Krause, Jens Christian Sørensen, Iben Lykke Petersen, Patrícia Duque-Estrada, Claudia Cappello, Ali Zein Alabiden Tlais, Raffaella Di Cagno, Lilit Ispiryan, Aylin W. Sahin, Elke K. Arendt, Emanuele Zannini

**Affiliations:** 1 School of Food and Nutritional Sciences, University College Cork, T12 YN60 Cork, Ireland; 2 SiccaDania A/S, Pilehøj 18, 3460 Birkerød, Denmark; 3 Department of Food Science, University of Copenhagen, 1958 Frederiksberg C, Denmark; 4 Facoltà di Scienze e Tecnologie, Piazza Università 5, 39100 Bolzano, Italy; 5 APC Microbiome Ireland, University College Cork, T12 YT20 Cork, Ireland; 6 Department of Environmental Biology, Sapienza University of Rome, Piazzale Aldo Moro 5, 00185 Rome, Italy

**Keywords:** plant protein, side-stream valorisation, resistant starch, food application, sustainability

## Abstract

Faba beans (*Vicia faba* L.) show exciting prospects as a sustainable source of protein and fibre, with the potential to transition to a more sustainable food production. This study reveals the compositional, nutritional and techno-functional characteristics of two protein isolates from faba beans (*Vicia faba* L.), a high-starch fraction and a high-fibre side-stream. During the analysis of those four ingredients, particular attention was paid to the isolates’ protein profile and the side-streams’ carbohydrate composition. The isoelectric precipitated protein isolate 1 showed a protein content of 72.64 ± 0.31% DM. It exhibited low solubility but superior digestibility and high foam stability. High foaming capacity and low protein digestibility were observed for protein isolate 2, with a protein content of 71.37 ± 0.93% DM. This fraction was highly soluble and consisted primarily of low molecular weight proteins. The high-starch fraction contained 83.87 ± 3.07% DM starch, of which about 66% was resistant starch. Over 65% of the high-fibre fraction was insoluble dietary fibre. The findings of this study provide a detailed understanding of different production fractions of faba beans, which is of great value for future product development.

## 1. Introduction

The world population is expected to reach almost 10 billion by 2050 [1]. The growing population places additional pressure on resources and interferes with the achievement of the Sustainable Development Goals (SDGs) proposed by the United Nations [2]. Primarily SDG No. 2, “end hunger”, and SDG No. 12, “ensure sustainable consumption”, are becoming more challenging due to the exploitation of resources. Furthermore, the changing climate makes crop failures and low harvest yields more likely [3], and global food security is in danger. As food production was responsible for more than a third of the global anthropogenic greenhouse gas emissions in 2015 [4], the need to find sustainable food solutions that meet the nutritional needs of the growing world population while adapting to changing climate conditions is increasing.

A shift to a higher percentage of plant-based foods could reduce greenhouse gas emissions and ensure the future supply of protein [5,6]. Pulses support a more sustainable diet [7,8,9], and faba beans (*Vicia faba* L.) are one of the most widely grown grain legumes [10]. Their production reduces greenhouse gas emissions compared to wheat monoculture and increases nitrogen fixation in the soil [11,12], and they provide plant protein, nutrients, dietary fibre and bioactive compounds [13].

Knowledge about the composition of ingredients is essential groundwork for new applications. By examining their techno-functional properties, the behaviour in a complex food matrix can be predicted. This study aims to analyse the composition and techno-functional properties of two faba bean protein isolates and two side-stream fractions of the protein isolation process.

The composition of different varieties of faba beans has been described by several authors in the past. Protein content between 22.70% and 35.66% of dry matter (DM) was reported in the beans [13,14], and fat content ranged from 0.70% to 2.00% DM. [13,15]. However, in addition to the starch content from 40.00% to 44.30% DM, information on the carbohydrate composition of faba beans is scarce. Therefore, this study focuses on providing more detailed information on the carbohydrate fractions of the studied protein isolates and side-stream fractions in addition to information on protein composition. In this regard, digestible and resistant starch and dietary fibre are determined.

Resistant starch is a nondigestible carbohydrate, therefore considered dietary fibre, and has high potential for treating gastrointestinal disorders [16]. Replacement of digestible starch with resistant starch reduces postprandial glycaemic response after meals and improves insulin response and microbiome diversity. 

Dietary fibre, which includes all types of nondigestible carbohydrates, has been proven to modulate the gut microbiota [17,18], which can stimulate the immune system and gut barrier function [19]. Depending on the physicochemical properties of dietary fibre, even affective processes and cognition can be positively influenced [18]. In this study, the total dietary fibre of the examined fractions is determined by the sum of insoluble and soluble dietary fibre. Knowing more about the structure and properties of the dietary fibre of these ingredients enables suitable application with targeted nutritional benefits.

As faba beans can cause favism, knowing the content of the antinutrients vicine and convicine is imperative. Studies [14,20,21] have reported the sum of vicine and convicine between 0.44 mg/g DM in zero-vicine varieties and up to 17.70 mg/g DM in other varieties. This study investigates the distribution of these two antinutrients, phytic acid, saponins and condensed tannins in the examined faba bean fractions and the effect of these antinutritional factors on the digestibility of protein isolates.

Furthermore, most articles that examined different fractions of faba beans focused on protein concentrates (56.4% DM protein) [22] or protein isolates (60.0–92.2% DM protein) [23,24,25]. Industrial production of these protein ingredients generates huge amounts of by-products that could be used as valuable ingredients [26]. More sustainable food production requires valorisation of side-streams; therefore, their potential for food applications must be determined beforehand.

In this study, to predict the behaviour of the ingredients in a food matrix, techno-functional properties are analysed. As the application of many plant proteins is limited by their solubility characteristics [27], this study evaluates the protein solubility of the two protein isolates at different pH values and relates this data to the surface hydrophobicity of these protein fractions. For all four fractions, the colour is measured because it plays an important role in the range of possible applications, particularly for meat and dairy alternatives [27]. When considering an application, liquid retention, such as oil and water holding capacities, is especially relevant in semi-solid foods, such as plant-based meats, eggs and yoghurt analogues [27].

In addition, knowledge about the foaming properties of ingredients is valuable, as it is desired for some food products, such as plant-based milk or ice cream. Excessive foaming, on the other hand, is undesirable for handling and bottling liquid products.

This article first presents the analysis of the composition of two faba bean protein isolates and two side-stream fractions of the protein isolation process, with a focus on their protein and carbohydrate profiles. Then, after evaluation of the compositional, nutritional, and techno-functional properties of the examined fractions, the accumulated data is linked with potential food applications. Including the side-stream fractions in the analysis will give new opportunities to include them in the production of foods and lead to a more sustainable production process.

## 2. Materials and Methods

### 2.1. Chemicals and Raw Materials

Unless otherwise stated, all chemicals were purchased from Sigma-Aldrich (St. Louis, MO, USA). SiccaDania A/S (Birkerød, Denmark) produced the protein isolates and their side-streams from commercial quality faba beans (*Vicia faba* L. cv. fuego).

### 2.2. Preparation of Faba Bean Protein Ingredients

Faba bean processing was performed by SiccaDania A/S at a pilot scale following a patented method [28], leading to the four fractions evaluated in this article. Protein isolate 1 (PI 1) was obtained by isoelectric precipitation at a pH of 3.5 or lower. The precipitate was reslurried, subjected to low-temperature pasteurisation at 50–80 °C and subsequently spray dried (SD900 SS, SiccaDania A/S, Denmark). Protein isolate 2 (PI 2) was extracted by membrane filtration from the soluble fraction after precipitation of PI 1 and spray dried. The side-stream of the protein isolation process was separated into a fibre-rich fraction (FRF) and a fraction high in starch (SF), and both products were dried using a zeta dryer (ZFD315, SiccaDania A/S, Denmark). The FRF was additionally passed through a 300 μm sieve after drying.

### 2.3. Compositional Analysis

The results of the compositional analysis were expressed as a percentage based on dry matter (DM). Moisture was determined according to AACC 44-17.01 by drying in an oven at 130 °C until weight was constant. The protein content was determined using the Kjeldahl method (AACC 46-12.01) with a nitrogen–protein conversion factor of 5.40 [29,30]. The fat content of the samples was determined using the Soxhlet method (AACC 30-25.01). Briefly, after a digestion step with 4 M HCl in a FOSS^®^ SC 247 SoxCap™ unit, fat was extracted with petroleum ether in a FOSS^®^ ST 243 Soxtec™ unit and gravimetrically determined. Digestible and resistant starch were analysed using the K-RAPRS enzyme assay kit (Megazyme, Bray, Ireland), based on McCleary et al. [31] (AOAC 2002.02, AACC 32-40.01). 

Sugars were extracted according to Hoehnel et al. [32] and determined with a limit of quantification for single sugars of 0.2 g/100 g DM by ion chromatography with pulsed amperometric detection using a gold electrode and a calibration against an internal standard. Fermentable short-chain carbohydrates (FODMAPs) were quantified with a limit of detection for single sugars of 0.005 g/100 g DM, based on Ispiryan et al. [33], as the sum of excess fructose, xylitol, sorbitol, mannitol, raffinose/stachyose and verbascose. 

Total dietary fibre was determined using the K-RINTDF enzyme assay kit (Megazyme, Bray, Ireland), based on AACC 32-60.01 and AOAC 2017.16, based on McCleary et al. [34], with slight modifications. After the incubation with pancreatic α-amylase and amyloglucosidase, the enzymes were inactivated at 95 °C. For the recovery of low molecular weight soluble dietary fibre (SDFS), the filtrates of the second filtration were concentrated by evaporating ethanol in a vacuum centrifuge (ScanVac, Lynge, Denmark) at 1500 rpm, 45 °C for 1 h and 2000 rpm, 50 °C for another 1.5 h. The volume of the concentrated samples was adjusted to 20 mL using 50 ppm sodium azide. Finally, an aliquot of 10 mL was deionised with 3 g of cation exchange (Amberlite^®^ FPA53 OH^−^) and 3 g of anion exchange (Ambersep^®^ 200H^+^). SDFS was determined using high-pressure liquid chromatography (HPLC), Agilent Infinity 1260 (Agilent Technologies Inc., Santa Clara, CA, USA), attached to a refractive index detector, based on the existing literature. 

### 2.4. Analysis of Antinutrients

#### 2.4.1. Vicine and Convicine

The contents of vicine and convicine in the faba bean ingredients fractions were analysed by capillary electrophoresis (CE) in a crude extract. Before extraction, 100 µL of a 62.0 mM 5-bromouridine internal standard were added to each sample. Five hundred milligrams of samples were extracted (3 × 1 min.) in 4 mL hot 70% methanol using an Ultra Turrax T 25 (Janke and Kunkel, Staufen, Germany). After evaporating the methanol, the sample was resuspended in 2 mL of ultrapure water. Micellar electrokinetic capillary chromatography (MECC) was used for CE with a buffer of 50 mM sodium dodecyl sulphate (SDS), 18 mM Na_2_B_4_O_7_, 30 mM Na_2_HPO_4_ and 5% 1-propanol at pH 7. The electrophoresis was performed on a 645 mm × 0.05 mm I.D. bare-fused silica capillary (Agilent Technologies, Santa Clara, USA) with an effective length of 560 mm, which was used at 17 kV at 20 °C for 25 min at a detection wavelength of 275 nm. The concentrations of vicine and convicine were calculated based on dry mass using an internal standard.

#### 2.4.2. Phytic Acid

Phytic acid concentrations were determined using the K-PHYT Phytic Acid (Phytate)/Total Phosphorus enzyme assay kit (Megazyme, Bray, Ireland), based on the AOAC method 986.11, following the manufacturer’s instructions [35].

#### 2.4.3. Total Saponin Content

The total saponin content (TSC) was quantified based on Lai et al. [36], with minor modifications to the extraction process, as reported by Nionelli et al. [37]. Briefly, 0.5 g of sample were defatted with 10 mL of petroleum ether by shaking for 4 h. After evaporating the solvent, 20 mg of the residues were used for extraction by shaking with 5 mL of 80% (*v/v*) aqueous methanol for 4 h. After centrifugation (9000× *g*, 10 min, at 4 °C, Thermo Scientific SL16R centrifuge), the supernatants were kept at 4 °C in the dark until analysis. Furthermore, 0.1 mL of the sample extract, 0.4 mL of 80% aqueous methanol, 0.5 mL of freshly prepared 8% ethanolic vanillin solution and 5.0 mL of 72% sulfuric acid were mixed in an ice-water bath. The mixture was heated at 60 °C for 10 min and then cooled in ice-cold water. Absorbance at 544 nm was measured against the reagent blank. The results were expressed as mg saponin/g extract from a calibration curve of saponin standard (CAS 8047-15-2) in 80% aqueous methanol.

#### 2.4.4. Condensed Tannins

Condensed tannins were determined using the vanillin assay, as described by Price et al. [38], with modifications. About 200 mg of sample material was extracted with 10 mL of absolute methanol for 20 min in rotating screw cap culture tubes. The mixture was centrifuged for 10 min at 3000× *g*, and the supernatant was used in the analysis. A six-point calibration (0–0.3 mg/mL) of catechin in absolute methanol was prepared. One millilitre portions of samples and standards were tempered to 30 °C in a water bath in duplicate. Meanwhile, the vanillin reagent was prepared by mixing one part 1% (*m/v*) vanillin in absolute methanol solution and one part 8% (*v/v*) concentrated HCl in absolute methanol solution. A total of 5 mL of preheated vanillin reagent was added to one set of sample and standard tubes, and 5 mL of preheated 4% aqueous HCl solution were added to 1 mL of the second set of tubes, maintaining 1 min intervals between additions. The samples were incubated for 20 min at 30 °C, removed, and the absorbance at 500 nm was read in 1 min intervals. Condensed tannins are expressed as catechin equivalents (CE) mg/g, based on the standard curve from the absorbance read at 500 nm against the reagent blanks.

#### 2.4.5. Trypsin and Chymotrypsin Inhibitor Activity

Trypsin inhibitor activity (TIA) was determined according to the American Oil Chemists’ Society (AOCS) Method Ba 12a-2020 [39] in three independent replicates, and each sample was measured in duplicate. One trypsin unit (TU) is defined as an increase of 0.02 absorbance at 410 nm under the 5 mL assay condition specified in the procedure. TIA is expressed as trypsin inhibitor units (TIU) per mg sample DM.

Chymotrypsin inhibition activity (CIA) was measured based on Alonso et al. [40]. Samples were extracted by stirring overnight with 0.05 M Tris-HCl buffer (pH 7.6) at a sample-to-buffer ratio of 1:10 (*w/v*). Fifty microlitres of the extracts were mixed with 100 μL of 0.005% chymotrypsin solution (in 0.05 M Tris-HCl buffer, pH 7.6), diluted to 1 mL with the same buffer. Next, a 2.5 mL portion of 0.001 M benzoyl-L-tyrosine ethyl ester (BTEE) was heated to 30 °C and added to the samples. Changes in absorbance were recorded at 256 nm directly after the addition of BTEE. Alonso et al. [40] defined one chymotrypsin unit as the increase by 0.01 absorbance unit at 256 nm of the reaction mixture.

### 2.5. Protein Analysis

Due to the low protein contents of SF and FRF, the protein profile, physicochemical traits, such as protein solubility and surface hydrophobicity, and protein digestibility were determined only on the protein isolates PI 1 and PI 2.

#### 2.5.1. Protein Solubility

Protein solubility at different pH values was evaluated in duplicate. Dispersions of 1% (w protein/v) in distilled water were prepared, and the pH value was adjusted. After shaking at 4 °C overnight, followed by centrifugation (4893× *g*, 20 min), the protein content of the supernatant was determined using the Kjeldahl method with a nitrogen-to-protein conversion factor of 5.40. Protein solubility was expressed as the percentage of the sample protein content in the dispersion remaining in the supernatant.

#### 2.5.2. Surface Hydrophobicity

The surface hydrophobicity of the protein isolates was analysed based on Hayakawa and Nakai, 1985 [41] with slight modifications [42]. First, protein dispersions were serially diluted with 0.01 M phosphate buffer (pH 7) in the range of 0.0006–0.015% (w protein/v). Then, 10 μL 1-anilino-8-naphthalene sulfonate (ANS) solution (8 mM in 0.1 M phosphate buffer, pH 7) were mixed with 4 mL of sample dilution and left in darkness at room temperature for 15 min. Fluorescence was measured at an excitation wavelength of 390 nm and an emission wavelength of 470 nm and corrected by a blank measured without adding ANS solution. The results were evaluated as the slopes (*R*^2^ ≥ 0.98) of the fluorescence intensity versus protein concentration (%) plot.

#### 2.5.3. Protein Profile

The protein profiles of the samples were analysed regarding their molecular weights using an Agilent Bioanalyzer 2100 Lab-on-a-Chip capillary electrophoresis system with an Agilent Protein 230 kit. The samples were prepared based on Amagliani et al. [43], with slight modifications. First, protein isolates were dispersed in an extraction buffer of 2% SDS, 2 M thiourea and 6 M urea to reach a protein concentration of 0.5%. After shaking on a platform shaker at room temperature for 2 h, the dispersions were centrifuged (3000× *g*, 30 min) to remove insoluble material. Dithiothreitol (DTT) was added to the sample buffer according to kit instructions for stronger reducing conditions.

#### 2.5.4. In Vitro Protein Digestibility

Simulated gastro–pancreatic in vitro protein digestibility (IVPD) was determined using a static, multistep digestion method [23,44]. Briefly, 50.0 ± 0.1 mg protein was used for digestion based on DM. Enzymatic hydrolysis consisted of pepsin digestion (1 h, 37 °C, pH 1–2) followed by short-term pancreatin digestion (+1 h, 37 °C, pH 7–8). Enzyme to substrate ratios (E:S) were maintained constant at 1:50 (*w/w*) for the pepsin digestion and 1:10 (*w/w*) for the pancreatin digestion. IVPD was calculated as the ratio between the concentration of free α-amino groups in the samples and the alanine standard solution, with the alanine standard representing 100% protein digestibility. A trinitrobenzene sulfonic acid (TNBS)-based method described by Joehnke et al. [45] was used to determine free amino groups.

### 2.6. Microscopy

Microstructural analysis was performed on a scanning electron microscopy (SEM). Faba bean fractions were mounted on aluminium stubs using carbon tape and sputter coated with a 5 nm gold–palladium layer (Au:Pd = 80:20) using a Polaron E 5150 sputter coating unit. Images were performed with a JEOL scanning electron microscope (JSM-5510, Jeol Ltd., Tokyo, Japan), operated at an accelerating voltage of 5 kV, a working distance of 20 mm and a magnification factor of 1000.

### 2.7. Techno-Functional Properties

#### 2.7.1. Colour Measurement

The colour of the faba bean fractions was analysed with a Chroma Meter (Minolta CR-300, Osaka, Japan) using the CIE L*a*b* colour space. The colour of samples was characterised by calculating the whiteness index (WI) [46]:

(1) $$WI\;\left[\%\right]=100-\sqrt{{\left(100-L^{*}\right)}^{2}+a^{*2}+b^{*2}}$$

The whiteness index was chosen for comparability and to describe the impact of the ingredients on food systems.

#### 2.7.2. pH and Total Titratable Acidity

The pH and the total titratable acidity (TTA) of the faba bean fractions’ dispersion in water (0.1 g/mL) were measured in duplicate on a digital pH meter (SevenGo Mettler Toledo Ag, Greifensee, Switzerland). TTA was determined using a modified version reported by Boeck et al. [47]. It is expressed as the volume of 0.1 M NaOH added per gram sample to adjust the pH to 8.5 and describes both the amount of free acids present and the sample’s buffering capacity.

#### 2.7.3. Foaming Properties

Foaming properties were examined according to Alonso-Miravalles et al. [48]. Dispersions of faba bean fractions of 2% (*w*/*v*) in ultrapure water were adjusted to pH 7, hydrated at 4 °C overnight, and frothed at room temperature using an Ultra-Turrax with an S10N-10G dispersing element (IKA-Labortechnik, Janke and Kunkel GmbH, Staufen, Germany) at maximum speed for 30 s. The heights of the liquid and foam phases of the samples were measured immediately and after 60 min. Foaming capacity describes the expansion of the sample at 0 min, while foam stability is expressed as sample expansion after 60 min as a percentage of sample expansion at 0 min. The expansion of the sample was calculated according to equation (2), where *SE* = sample expansion, *h_i_* = initial sample height and *h_t_* = sample height at measured time:

(2) $$SE\;\left[\%\right]=\frac{h_{t}-h_{i}}{h_{i}}\cdot 100$$

#### 2.7.4. Water and Oil Binding Capacity

Water and oil binding capacities were determined based on Boye et al. [49] (AACC 56-11.02), with slight modifications. Water binding capacity (WBC) was expressed in percent (*w/w*) as the amount of water absorbed by 1 g of the sample after mixing for 3 min with a vortex mixer (VM-10, DAIHAN Scientific, Wonju-si, South Korea), followed by 1 h of waiting time. Oil binding capacity (OBC) describes the weight of oil retained by 1 g of the faba bean fraction after the same treatment.

#### 2.7.5. Statistical Data Analysis

Unless otherwise stated, the analyses were performed in triplicate. Data was tested for normality, and results were analysed using a one-way analysis of variance (ANOVA) followed by Tukey’s post hoc test (*p* < 0.05), unless otherwise specified. When equal variances were not assumed, a correction using Welch and Games–Howell post hoc test (*p* < 0.05) was applied. All statistical analyses were conducted using GraphPad Prism 5.00 Software, San Diego, CA, USA, and IBM SPSS Statistics for Windows, Version 28.0.0.0, Chicago, IL, USA. Correlation analysis was carried out using Microsoft Excel, Version 2211, Redmond, WA, USA.

## 3. Results and Discussion

### 3.1. Compositional Analysis

Table 1 shows the nutritional composition of the four fractions of faba beans investigated. When comparing the protein contents of the two protein isolates, PI 1 and PI 2, there was no significant difference between the two samples. The protein content of isoelectric precipitated faba bean isolates has been reported to be 80–90% DM and higher [23,25,50]. The significantly higher protein content reported in the literature can be attributed to different protein conversion factors. While 6.25 is commonly used as a nitrogen-to-protein conversion factor in the literature, Mariotti et al. [29] and Mossé [30] recommend a conversion factor of 5.40 for pulses based on amino acid analysis and their nitrogen-to-protein response. If the different protein conversion factors are considered, no significant differences can be observed between the protein contents of PI 1 and PI 2 and the values previously reported for faba bean protein isolates.

Fat contents have been reported from 1.53% DM to 2.02% DM [13,22,50] in faba bean seeds. Therefore, it seems that fat is concentrated in PI 1, which showed a fat content of 6.24 ± 0.18% DM, while only small amounts of fat were found in PI 2. As PI 1 is produced by precipitation at the isoelectric point, other compounds from faba beans may not be completely separated but may precipitate with the protein. Ray and Georges [51] reported large amounts of oleosins in faba beans. Oleosins are fat-associated proteins of about 15–26 kDa that are found in seed oil bodies, stabilising their surface and preventing intracellular lipid droplets from coalescing [52]. Coprecipitation could also explain the significantly higher amount of starch detected in PI 1 compared to PI 2. As PI 2 is obtained from the soluble fraction after the precipitation, fewer carbohydrates remain in PI 2. Nevertheless, both protein ingredients contain relatively low amounts of resistant and digestible starch.

**Figure 1:**
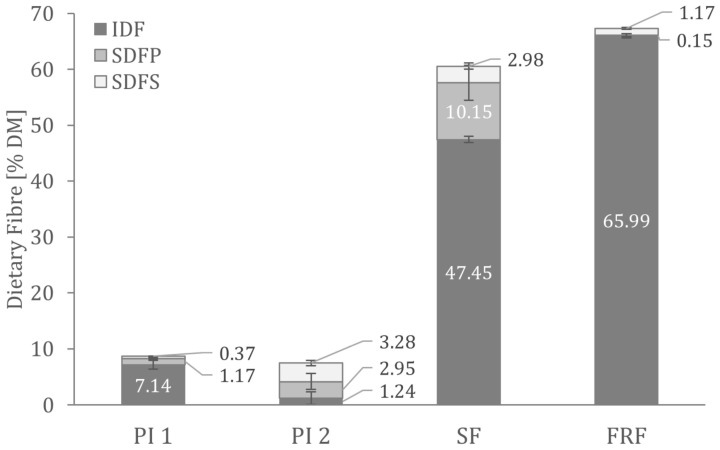
Dietary fibre composition in the analysed faba bean fractions. IDF—insoluble dietary fibre; SDFP—high molecular weight soluble dietary fibre; SDFS—low molecular weight soluble dietary fibre.

Figure 1 shows the dietary fibre composition analysed in the faba bean fractions. There were no significant differences in the total dietary fibre content of the two protein isolates. As predicted from the production processes, the fibre content of PI 1 consists primarily of insoluble dietary fibre. In contrast, the PI 2 fibre fraction comprises more than 80% soluble dietary fibre.

In conclusion, PI 1 and PI 2 are both valuable protein ingredients. However, PI 1 contains higher percentages of fat and starch than PI 2. The higher fat content of PI 1 could enhance flavours and decrease bitterness in food applications [53]. However, PI 2 seems to be the purer protein ingredient that can be used in applications where only protein is intended to be added, e.g., for low-fat and low-carb products.

As expected, the side-stream fractions showed significantly lower protein contents than the protein isolates. At the same time, the purification process of the side-streams retained the remaining protein in the fibre-rich fraction. As a result, SF is a highly pure product, with no fat and only traces of protein.

In SF, more than 60% of the total starch is resistant starch. Resistant starch is considered a dietary fibre and is associated with beneficial effects on the gut microbiome and colon health [54]. As a result of the high amount of resistant starch, SF contains 62.28 ± 0.76% DM dietary fibre, which consists of approximately 89% resistant starch.

Other than high amounts of dietary fibre, FRF contains about 15% resistant starch. Analysis showed that insoluble fibre contributes the most to total dietary fibre in FRF (see Figure 1).

In applications, SF could be used as a replacement for wheat starch. The high content of resistant starch in SF can enhance the nutritional quality of a product by adding dietary fibre. Therefore, the FRF could also be suited for application in high-fibre bakery products. In addition, the high amount of protein and dietary fibre together might improve the satiety of consumers while reducing the energy density of foods and increasing stool bulk [55,56].

### 3.2. Antinutritional Factors

The results of the analysis of the main antinutritional compounds in the faba bean fractions can be found in Table 2. 

#### 3.2.1. Vicine and Convicine

Of the four fractions of faba beans, vicine and convicine could only be detected in PI 1 and PI 2. Those two pyrimidine glycosides are precursors for the main factors of favism, a genetic disease characterised by the lack of the enzyme glucose-6-phosphate dehydrogenase. Consumption of faba beans with high vicine and convicine content could cause severe and potentially lethal haemolytic anaemia in affected individuals [15,57,58]. Although vicine and convicine are only slightly soluble at neutral pH values [59], the solubility of both substances increases significantly in dilute alkalis and acids [60]. Due to the low pH during isoelectric precipitation, it was expected that vicine and convicine remained in solution and did not precipitate with PI 1. This hypothesis could be confirmed as vicine was not found in PI 1, and convicine contents were very low. The low convicine content results from the lower solubility of convicine compared to vicine in acidic conditions [59,61]. As both glycosides were detected in low amounts, it can be assumed that the isoelectric precipitation removes these antinutrients well. The literature [14,20,21] reported the content of vicine and convicine for *Vicia faba* L. cv. fuego between 2.1 mg/g and 7.73 mg/g for vicine and between 2.37 mg/g and 8.1 mg/g for convicine. All faba bean fractions reported here show vicine and convicine contents in the lower range or below the previously published values, indicating that the extraction process successfully reduced vicine and convicine.

#### 3.2.2. Phytic Acid

Phytic acid, which forms complexes with proteins and critical minerals and therefore inhibits protein digestion, has previously been reported between 0.83 g/100 g and 3.25 g/100 g in faba beans [13,50,62]. The four examined faba bean fractions show significantly lower phytic acid contents than in the literature. However, as phytic acid binds to the protein fraction of faba beans [63], significant differences can be seen between the fractions analysed. A correlation of *r* = 0.76 (*p* < 0.05) could be determined between protein content and condensed tannin amounts. Nutritionally, no adverse effect was expected by phytic acid contents of all four fractions. Different additional treatments of faba beans and the obtained fractions, such as soaking, dehulling, fermentation and heat treatments, showed promising results for reducing phytic acid and other antinutrients [62,64,65].

#### 3.2.3. Total Saponin Content

Mayer Labba et al. [14] reported saponin contents in different faba bean cultivars between 0.02 mg/g and 0.11 mg/g. Saponins are heat-stable water-soluble compounds containing a steroid or triterpenoid aglycone linked to one or more oligosaccharides [66]. They induce a bitter taste in high concentrations, reduce nutrient bioavailability and decrease trypsin and chymotrypsin activity [67]. However, along with their nutritionally adverse effects, several health benefits have been associated with saponin consumption, such as anti-inflammatory, antimicrobial, immunostimulant, hypocholesterolaemic and anticarcinogenic effects [67,68,69]. While neither protein isolate contains saponins, and SF shows negligible amounts of them, FRF displays a higher saponin content. As some of the sugars in saponins are β-1-4-linked, they would be considered soluble dietary fibre [70]. During the protein isolation process, saponins stay in the soluble phase, as most of them are highly water-soluble [71]. The separation of the protein isolates and side-streams seems to concentrate saponins in the FRF.

#### 3.2.4. Condensed Tannins

Tannins are heat-stable polyphenols that reduce protein digestibility by either making protein partially unavailable or inhibiting digestive enzymes and increasing faecal nitrogen [67]. According to Ivarsson et al. [21], comparing tannin results from different studies is difficult due to their structural complexity and differences in analytical methods. However, of the four faba bean fractions, it can be observed that no tannins were found in PI 2 and only negligible amounts in FRF. On the other hand, PI 1 and SF exhibited the highest condensed tannin content among the four fractions tested. This could be explained by the formation of tannin–protein complexes [65] and precipitation along with the PI 1 fraction. Additionally, tannins have been found to interact with amylose and linear fragments of amylopectin, suggesting hydrophobic interactions [72].

#### 3.2.5. Trypsin and Chymotrypsin Inhibitor Activity

Lastly, the overall trypsin and chymotrypsin inhibition has also been determined as an important component of the antinutrient factors. In the literature [6,17,19,30,32,35], trypsin inhibition in faba beans is reported to between 1.2 TIU/mg and 23.10 TIU/mg. Mayer Labba et al. [30] reported 13.70 TIU/mg for *Vicia faba* L. cv. fuego. As shown in Table 2, PI 1 exhibits low TIA compared to the literature. At the same time, it was detected as high in PI 2; one explanation for this could be that the trypsin inhibitors described in legumes generally have a smaller molecular weight and are highly water soluble [73,74]. Due to the isolation process, smaller and more water-soluble proteins are concentrated in PI 2 (see Section 3.3.2). To improve the nutritional value, prolonged thermal treatment or other processing methods could reduce the TIA in PI 2 before consumption [73,75].

The other three faba bean fractions showed overall low TIA. As SF showed a very low protein content, trypsin inhibitors were not detected. TIA in FRF was detected in low amounts. Differences from values of TIA in the literature might be due to different methodology as well as different TIA in different cultivars and agronomic conditions [76].

In addition to trypsin inhibition, chymotrypsin inhibition plays an essential role in affecting protein digestibility [66]. As no official and standardised method for chymotrypsin inhibition activity is available, comparing results between different studies is not feasible [77]. Comparing the chymotrypsin inhibition of the four fractions, PI 2 shows the highest chymotrypsin inhibition out of the faba bean fractions. Ye et al. [78] described a Bowman–Birk-type trypsin–chymotrypsin inhibitor in faba beans with a molecular mass of 7.5 kDa. As PI 2 consists mainly of smaller proteins, this inhibitor peptide or similar ones could be concentrated in that fraction. A strong correlation of *r* = 1.00 (*p* < 0.05) was determined between trypsin and chymotrypsin inhibitor activities. No significant difference between the other three samples could be found in the chymotrypsin inhibition activity.

Regarding the antinutritional factors in the examined faba bean fractions, the high saponin content in FRF and its possible sensory effects, condensed tannins in PI 1 and SF and the trypsin and chymotrypsin activities of PI 2 should be considered when prototyping foods with those ingredients.

### 3.3. Protein Analysis

#### 3.3.1. Protein Solubility and Surface Hydrophobicity

**Figure 2:**
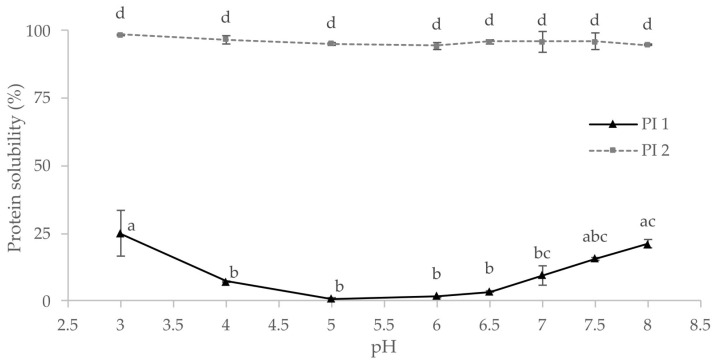
Protein solubility of the protein isolates PI 1 and PI 2 at different pH values; same letters indicate no significant differences (*p* < 0.05).

Knowledge of a protein’s techno-functional properties and solubility is essential for formulating plant-based foods [27]. Protein solubility is required for many functional properties such as emulsification, foaming, or gelling [23,79]. As illustrated in Figure 2, protein solubility at different pH values has been determined for the protein isolates PI 1 and PI 2. As predicted from the production process, PI 1 generally showed lower solubility than PI 2. There was no significant difference between the solubilities of PI 2 at different pH values, but the solubility of PI 1 depended on the pH.

**Figure 3:**
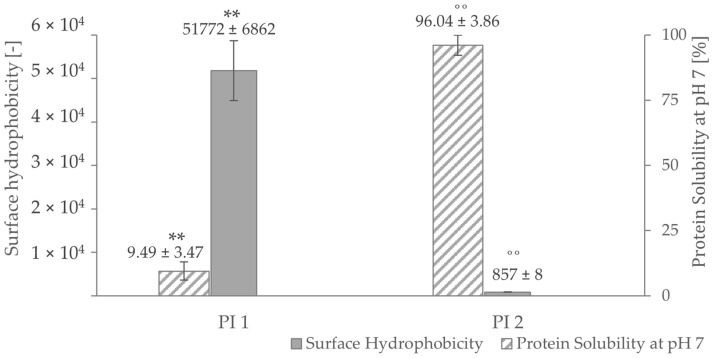
Protein solubility and surface hydrophobicity at pH 7 of the protein isolates PI 1 and PI 2. ** negative correlation with a Pearson correlation coefficient of *r* = 0.81 (*p* < 0.05). ˚˚ negative correlation with a Pearson correlation coefficient of *r* = 1.00 (*p* < 0.05).

High surface hydrophobicity increases the tendency of proteins to aggregate [80] and affects their solubility. As pictured in Figure 3, PI 1 shows a very high surface hydrophobicity, but it is scarcely soluble at pH 7. On the other hand, a low protein surface hydrophobicity was measured for PI 2, which is highly soluble at pH 7. A strong correlation of *r* = 0.81 (*p* < 0.05) for PI 1 and *r* = 1.00 (*p* < 0.05) for PI 2 could be determined between surface hydrophobicity and protein solubility at pH 7. The difference in surface hydrophobicity of the two isolates could be explained by the different processes of retaining those fractions. Usually, hydrophobic residues tend to be buried inside the core of a protein [81,82,83]. By denaturation, which happens during the isoelectric precipitation process of PI 1 [84], the hydrophobic regions of PI 1 are more exposed than the ones of PI 1, leading to a higher measured surface hydrophobicity [23]. Additionally, the difference in surface hydrophobicity between the two isolates could be due to difference in amino acid composition in both protein fractions.

While a high surface hydrophobicity is usually undesired because of low solubility and the tendency of proteins to aggregate, this does not necessarily have a negative impact on the application functionality of PI 1. Majzoobi et al. [85] showed that even mostly water-insoluble plant proteins, such as glutelin and prolamin, could still be hydrated and bind considerable amounts of water. Therefore, PI 1 might be very suitable for application in a solid matrix, such as bread or a plant-based meat alternative. For meat alternatives in particular, it is not necessary to focus on protein purity and high protein solubility [86].

In contrast, PI 2 might be considered in beverage applications, such as plant-based drinks, where high solubility is required to avoid precipitation or a sandy mouthfeel.

#### 3.3.2. Protein Profiles

**Figure 4:**
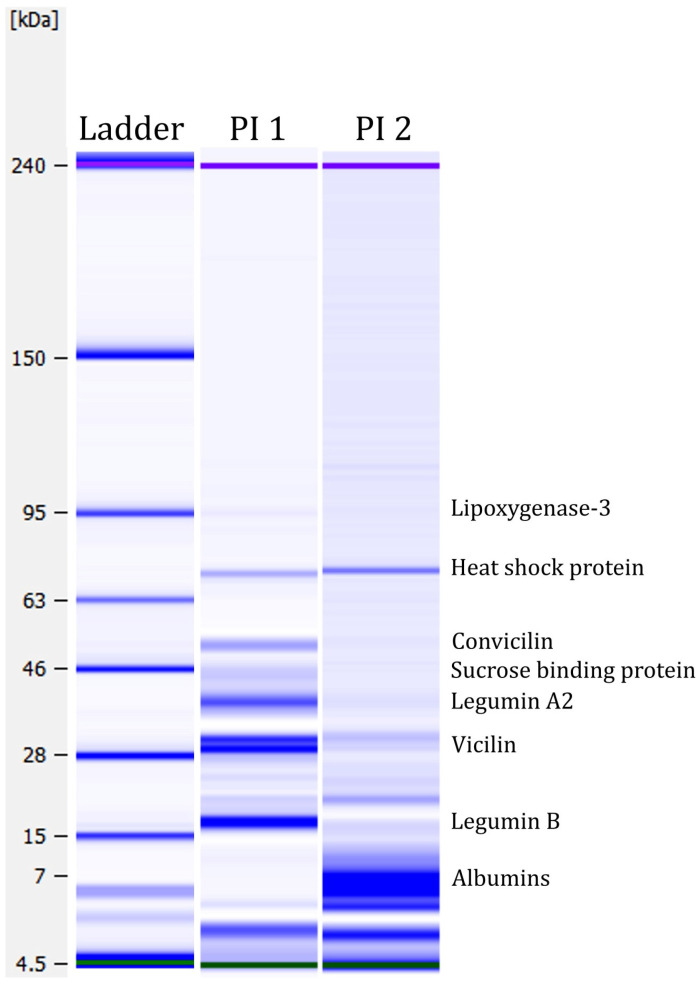
Protein profiles under strong reducing conditions measured with the Agilent Bioanalyzer. Green and purple bands are marker bands. Protein assignment, according to Warsame et al. [87].

Lab-on-a-Chip capillary electrophoresis was performed to analyse the protein profiles of the two protein isolates. Figure 4 shows the protein profiles under strong reducing conditions. The figure shows several protein bands. A weak protein band was detected in PI 1 at around ~95 kDa, which could correspond to the seed linoleate 9S-lipoxygenase-3 [87]. Lipoxygenase is antinutritional as it participates in lipid oxidation and generates undesirable flavours in applications [88]. By deactivating lipoxygenase before processing, these antinutritional properties could be minimised. A strong protein band was detected at ~55 kDa in PI 1 but not in PI 2. This band might be the globulin convicilin [87]. The trimeric protein is one of the primary storage proteins in *Vicia faba* L. [89]. The protein bands detected at ~40 kDa and ~20 kDa in PI 1 could correspond to the globulin legumin [87]. Legumin is a major protein in faba bean seeds, representing approximately 50% of storage proteins [90]. Legumin consists of six subunit pairs of an acidic (~40 kDa) and a basic (~20 kDa) part, linked by a single disulphide bond [91]. At ~30 kDa, two strong protein bands were detected in PI 1. In PI 2, only weak bands were visible. These protein bands could correspond to vicilin, which is the third main globulin storage protein in faba beans. Both vicilin and convicilin are the main potential food allergens in the seeds of the *Viciae* family [92]. Furthermore, Warsame et al. [87] identified the 70 kDa heat shock protein in faba beans, which could be the protein band detected at ~70 kDa in PI 1 and PI 2. They also described a sucrose-binding protein, which could potentially explain the band at ~45 kDa in PI 1. PI 2 demonstrates mainly smaller proteins, between 7 and 10 kDa. Those protein bands could correspond to albumins, highly water-soluble globular proteins that experience heat denaturation [87,93].

#### 3.3.3. In Vitro Protein Digestibility

In vitro protein digestibility (IVPD) of the protein isolates was determined after pepsin and pancreatin digestion, mimicking digestibility in the human upper digestive tract. The results of the IVPD analysis of the two protein isolates are shown in Table 3. Peptide bonds degraded during the gastric phase are indicated by Pepsin digestibility (1 h). Total digestibility (2 h) describes the overall rate of degraded peptide bonds after the combined stages of gastric and pancreatic digestion. As a reference protein, bovine serum albumin (BSA) is known to be a highly digestible protein [45].

It can be observed that PI 1 exhibits significantly (*p* < 0.05) higher total protein digestibility than PI 2. This could be traced back to the antinutritional factors, as PI 2 showed higher trypsin + chymotrypsin inhibition (221.34 ± 13.11%) than PI 1 (4.37 ± 0.80%). Although Longstaff et al. [94] noted that high tannin contents inhibit trypsin digestion, the higher content of condensed tannins does not seem to negatively affect the digestibility of PI1. On the contrary, no significant differences were detected when comparing the total protein digestibility of PI 1 with the reference BSA. Therefore, PI 1 can be considered highly digestible. It can be assumed that the difference in digestibility of the two isolates is influenced more by the protein structure and profile than by single antinutritional compounds. The high surface hydrophobicity of PI 1 in particular is beneficial for high digestibility, as this indicates a more unfolded protein structure resulting in higher accessibility to digestive enzymes [95,96].

### 3.4. Microscopy

**Figure 5:**
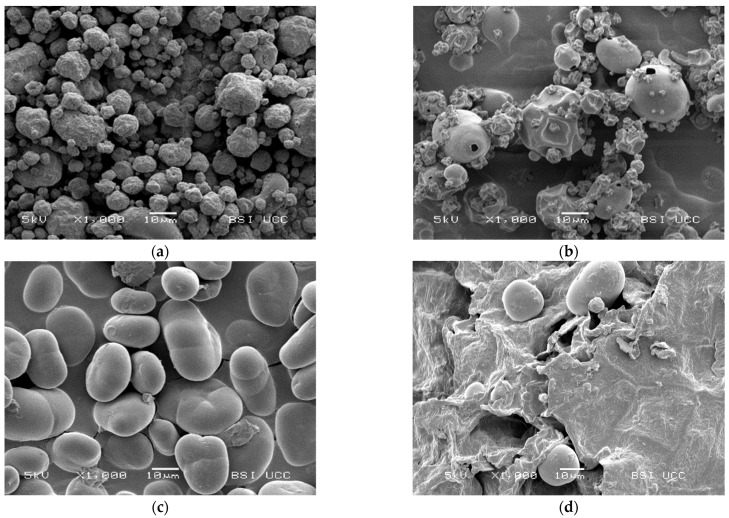
Scanning electron microscopy (SEM) pictures of the examined faba bean fractions: (**a**) PI 1, magnification factor = 1000; (**b**) PI 2, magnification factor = 1000; (**c**) SF, magnification factor = 1000; (**d**) FRF, magnification factor = 1000.

The SEM pictures in Figure 5 show the different morphology of the examined faba bean fractions. PI 1 is characterised by a homogeneous profile of small globular protein particles of ~5–20 μm. The shrivelled surface and shrunken particles are typical for spray-dried protein powders and result from rapid moisture evaporation during the drying process [97,98,99].

On the other hand, the morphology of PI 2 seems to be much more inhomogeneous. Some larger, round particles of ~20 μm can be found between small protein particles of up to 10 μm. These particles show a smooth surface with holes in them; some of them are also shrunken. The holes and shrunken surfaces could result from high-temperature drying [99]. SF shows a typical picture of faba bean starch granules and small cell wall residues. Figure 5c also shows the previously reported cracks in the starch granules [100]. According to Blennow et al. [101] and Glaring et al. [102], those cracks may reflect low granule integrity due to suboptimal packing of starch chains within the granules. Figure 5d shows large fibre particles in FRF with a rough and uneven surface and few starch granules between them. This observation is similar to Saldanha do Carmo et al. [24], who remarked that other cellular structures than proteins remained larger in faba beans, even after milling.

### 3.5. Techno-Functional Properties

#### 3.5.1. Colour Measurement

As described in Table 4, all four faba bean ingredients exhibit a whiteness index (WI) of over 80%. SF is almost white with a whiteness index of 96.52%, while the other three fractions show colours within the beige to light brown spectrum. Out of the analysed samples, FRF is the darkest, with the two protein isolates being slightly lighter. As FRF includes a minor amount of faba bean hulls, the darker colour could originate from the darker faba bean hulls [103]. Overall, the differences in whiteness index between samples are minimal, making all four faba bean fractions suited for most food applications as they do not cause discolouration.

#### 3.5.2. pH and Total Titratable Acidity

When analysing an ingredient’s acidity, two values should be considered. First, the pH of the component in water shows the immediate effects of the ingredient on the food matrix. Additionally, total titratable acidity (TTA) considers the buffering capacity of the ingredient and the soluble components at specific pH values. Although both protein isolates exhibit a slightly acidic pH due to the isoelectric precipitation and a high buffering capacity (see Table 4), much lower buffering capacities are observed in the side-stream fractions SF and FRF. Other than peptides, amino acids and organic acids, proteins are the most important compounds affecting the buffering capacity in foods [104], which explains the significantly higher TTAs of the protein isolates compared to the side-stream fractions. A high buffering capacity can be beneficial in food applications to prevent protein aggregation at their isoelectric point as the pH remains more stable [105]. The initial pH levels of FRF are neutral, whereas the initial pH levels of SF are slightly basic. Based on the pH and TTA measurements, the two protein isolates might exhibit a faintly acidic taste in food applications. As the side-stream’s pH values are closer to neutral, no adverse effect on food products is expected.

#### 3.5.3. Foaming Properties

When comparing the techno-functional properties of the four faba bean fractions, as shown in Table 4, it is noticeable that PI 2 showed extraordinary foaming capacity (FC) with moderate foam stability (FS). On the other hand, PI 1 forms very stable foams. Statistically, a strong correlation could be determined between the protein surface hydrophobicities and foaming capacities (Pearson correlation coefficient *r* = −0.97, *p* < 0.05) and foaming stabilities (*r* = −0.94, *p* < 0.05) of the two protein isolates.

While proteins generally act as emulsifiers stabilising foams [106], the protein profile in the two isolates is vastly different. As described before, PI 1 consists mainly of globular and higher molecular weight proteins, while a large part of PI 2 consists of lower molecular weight albumins, which are heat instable. Amagliani and Schmitt, 2017 [107] and Munialo et al. [108] reported the formation of micrometre-long fibrillar protein aggregates after heat treatment that could stabilise foams and improve emulsifying properties. Furthermore, Makri et al. [109] described an improvement in foamability and a slight reduction in foam stability by albumins. However, since the foaming analysis was performed in an aqueous matrix, the low foaming capacity of PI 1 could also result from its low solubility in water. Although foaming is desired for food products, such as whippable creams, ice cream or egg analogues [27], excessive foaming may be undesirable for handling and bottling liquid products.

Regarding the side-stream fractions, FRF forms slight foams with no foam stability, but SF does not create foam. These results reflect the expected outcome of this analysis, as foam-stabilising properties are mainly attributed to matrices high in protein or fat.

#### 3.5.4. Water and Oil Binding Capacities

Knowing the water and oil binding capacities of ingredients is especially relevant in semisolid foods, such as plant-based meats, egg and yoghurt analogues. Retention of these fluids is critical in their perceived juiciness [110], and the separation of liquids might be visibly unappealing [111]. The OBC of protein ingredients is of great interest for applications, as it is reflected in the emulsifying capacity, which is relevant for products such as mayonnaise [112].

Table 4 shows the WBC and OBC of the faba bean ingredients. PI 1 exhibited a high WBC and a moderate OBC. On the other hand, the OBC of PI 2 was more than three times higher than PI 1, which indicates much better emulsification properties of this ingredient.

A high OBC was expected for PI 1 due to its high surface hydrophobicity [113]. According to Kinsella et al. [114] and Sathe et al. [115], fat is bound to the nonpolar side chains of proteins. Additionally, oil binding capacity is closely related to the physical entrapment of oil [113,116]. Therefore, protein powders with a low-density and a small particle size adsorb and entrap more oil than high-density protein powders do [113]. As PI 2 consisted of smaller particles than PI 1 and had a lower density, this could explain the difference in OBC determined between the two samples.

In the side-streams, SF had relatively low water and oil binding capacities. As the determination of WBC and OBC does not include a heating step, the WBC does not correspond to starch gelatinisation but only to the trapping of liquids in the starch granular pores and channel structure [117], which is minimal in intact starch granules of SF.

In contrast to those results, FRF showed a remarkably WBC and a very high OBC. According to Blackwood et al. [118], the WBC of a fibre depends mainly on the chemical properties and physical structure, as insoluble fibres retain water in a network of pores. Therefore, the high WBC of FRF is a combined effect of hydroxyl groups on its surface, interacting with water molecules through hydrogen bonding [119] and its porous surface, which could be observed in the SEM pictures.

## 4. Conclusions

Overall, the results of this study showed that the four investigated fractions of faba beans have promising properties for future applications. Each ingredient possesses valuable characteristics for its complete integration into the food chain. PI 1 and PI 2 are valuable protein ingredients. The different conditions in their isolation processes are reflected in the techno-functional properties.

The isoelectric precipitation concentrated larger and fewer water-soluble proteins in PI 1, which displayed excellent digestibility and high foam stability. Due to the low solubility of PI 1, the most suitable applications for PI 1 might be in a solid matrix, such as bread or meat alternatives.

The soluble fraction, from which PI 2 was isolated, consists of smaller proteins and exhibited high foaming capacity but lower digestibility. PI 2 could be utilised in a foamable high-protein plant-based drink.

The SF contained 83.87% starch, of which 66.06% was resistant starch. Due to this high proportion of indigestible carbohydrates, the SF wheat could be used as a replacement for the currently ubiquitous wheat and corn starch in foods, such as desserts or sauces, leading to both a reduction in the glycaemic index and improved intestinal health.

The FRF is characterised by over 65% of insoluble dietary fibre, a relatively high saponin content and a very high fluid absorbency. While these properties need to be carefully considered in recipe development, the FRF could play a vital role as a fat replacer or bulking agent in food products.

All four fractions showed a neutral colour, enabling them to be easily integrated into potential new food products.

These results provide a significant first step towards the use of the examined faba bean fractions in a wider range of applications, broadening their potential from their current primary use as feed to valuable ingredients in novel food development. Identification of applications for the carbohydrate-rich side-streams in the protein isolation process not only improves cost effectiveness but also leads to a more sustainable food production, enabling the UN’s Sustainable Development Goals. Future research should investigate the practical suitability of those ingredients in the suggested food products.

## Figures and Tables

**Table 1:** Compositional analysis of faba bean fractions, average contents ± standard deviation (g/100 g DM).

	PI 1	PI 2	SF	FRF
Protein	72.64 ± 0.31 ^a^	71.37 ± 0.93 ^a^	0.18 ± 0.01 ^c^	16.70 ± 0.08 ^b^
Fat ^1^	6.24 ± 0.18 ^a^	0.78 ± 0.04 ^b^	n.d.	n.d.
Digestible Starch	5.32 ± 0.28 ^c^	1.00 ± 0.07 ^d^	28.46 ± 0.37 ^a^	15.06 ± 0.22 ^b^
Resistant Starch ^2^	0.33 ± 0.01 ^c^	n.d.	55.41 ± 3.31 ^a^	9.70 ± 0.44 ^b^
Σ Total Starch	5.65 ± 0.27 ^c^	1.00 ± 0.07 ^d^	83.87 ± 3.07 ^a^	24.76 ± 0.61 ^b^
Total Dietary Fibre	8.69 ± 0.67 ^c^	7.64 ± 1.53 ^c^	62.28 ± 0.76 ^b^	69.09 ± 0.24 ^a^
FODMAPs ^3^	n.d.	0.07 ± 0.00 ^a^	0.06 ± 0.00 ^a^	n.d.

Sugars were not detected or were at levels below 0.02 g/100 g DM. Different letters indicate significant difference (*p* < 0.05). ^1^ n.d., not detected. ^2^ n.d., not detected or below 0.09 g/100 g DM. ^3^ n.d., not detected or below 0.025 g/100 g DM.

**Table 2:** Antinutritional factors of the faba bean fractions, means ± standard deviation.

	PI 1	PI 2	SF	FRF
Vicine (mg/g DM)	n.d. ^1^	2.18 ± 0.11 ^a^	n.d. ^1^	n.d. ^1^
Convicine (mg/g DM)	0.30 ± 0.03 ^a^	1.52 ± 0.19 ^b^	n.d. ^1^	n.d. ^1^
Phytic acid (g/100 g DM)	0.39 ± 0.04 ^a^	0.14 ± 0.02 ^b^	0.008 ± 0.011 ^c^	0.16 ± 0.01 ^b^
Saponins (mg/g DM)	n.d. ^1^	n.d. ^1^	0.97 ± 0.08 ^a^	4.94 ± 0.08 ^b^
Condensed tannins (CE mg/g DM)	7.19 ± 1.45 ^a^	n.d. ^1^	3.23 ± 0.54 ^a^	0.55 ± 0.00 ^b^
Trypsin inhibitors (TIU/mg DM)	2.72 ± 0.04 ^a^	206.73 ± 12.01 ^b^	n.d. ^1^	3.38 ± 0.08 ^c^
Chymotrypsin inhibitors (CIU/mg DM)	1.65 ± 0.76 ^a^	14.61 ± 1.11 ^b^	0.85 ± 0.10 ^a^	1.63 ± 0.07 ^a^

One-way analysis of variance (ANOVA) followed by Tukey’s post hoc test was carried out within each row; different letters indicate significant difference (*p* < 0.05). ^1^ n.d., not detected.

**Table 3:** In vitro protein digestibility (IVPD) (%) of protein isolates according to the stage of digestion over short-term digestion.

Protein Isolates	Pepsin Digestibility (1 h)	Total Digestibility (2 h)
PI 1	5.31 ± 0.03 ^a^	23.30 ± 1.18 ^a^
PI 2	1.98 ± 0.29 ^b^	9.59 ± 0.13 ^a^
BSA (reference)	5.75 ± 0.11 ^c^	23.85 ± 0.15 ^a^

The results are shown as mean ± standard deviation. Different letters within the same column indicate significant differences (*p* < 0.05). Total digestibility = pepsin (1 h) + pancreatin (1 h) protein digestibility in the short term. BSA: bovine serum albumin.

**Table 4:** Techno-functional properties of faba bean fractions, values ± standard deviation.

		PI 1	PI 2	SF	FRF
WI	(%)	81.21 ± 0.95 ^c^	88.10 ± 0.21 ^b^	96.52 ± 0.50 ^a^	80.21 ± 0.74 ^d^
pH	(-)	5.14 ± 0.04 ^c^	4.57 ± 0.00 ^d^	8.02 ± 0.06 ^a^	6.36 ± 0.02 ^b^
TTA	(mL/g)	4.80 ± 0.05 ^b^	6.67 ± 0.55 ^a^	0.02 ± 0.00 ^d^	0.45 ± 0.01 ^c^
FC	(%)	18.06 ± 2.41 ^b^	133.33 ± 15.73 ^a^	0.00 ± 0.00 ^d^	7.92 ± 1.34 ^c^
FS	(%)	70.00 ± 8.66 ^a^	39.79 ± 4.09 ^b^	- ^2^	0.00 ± 0.00 ^c^
WBC	(%)	149.80 ± 0.61 ^b^	- ^1^	63.67 ± 1.28 ^c^	1028.92 ± 56.90 ^a^
OBC	(%)	65.32 ± 0.33 ^c^	229.13 ± 4.42 ^b^	49.43 ± 1.55 ^d^	342.35 ± 4.96 ^a^

WI—whiteness index; TTA—total titratable acidity; FC—foaming capacity; FS—foam stability; WBC—water binding capacity; OBC—oil binding capacity; results referred to DM. Different letters indicate significant differences (*p* < 0.05). ^1^ No analysis of water binding capacity was carried out on PI 2 due to high solubility. ^2^ No analysis of foam stability due to no foaming capacity.

## Data Availability

Data are contained within the article.
